# A method for genotype validation and primer assessment in heterozygote-deficient species, as demonstrated in the prosobranch mollusc *Hydrobia ulvae*

**DOI:** 10.1186/1471-2156-9-55

**Published:** 2008-08-19

**Authors:** Robert J Brownlow, Deborah A Dawson, Gavin J Horsburgh, James J Bell, John D Fish

**Affiliations:** 1 NERC Molecular Genetics Facility, Department of Animal and Plant Sciences, University of Sheffield, Sheffield, S10 2TN, UK; 2 Institute of Biological Sciences, Aberystwyth University, Wales, SY23 3DA, UK; 3 Centre for Marine Environmental and Economic Research, School of Biological Sciences, Victoria University of Wellington, Po Box 600, Wellington, New Zealand

## Abstract

**Background:**

In studies where microsatellite markers are employed, it is essential that the primers designed will reliably and consistently amplify target loci. In populations conforming to Hardy-Weinberg equilibrium (HWE), screening for unreliable markers often relies on the identification of heterozygote deficiencies and subsequent departures from HWE. However, since many populations naturally deviate from HWE, such as many marine invertebrates, it can be difficult to distinguish heterozygote deficiencies resulting from unreliable markers from natural processes. Thus, studies of populations that are suspected to deviate from HWE naturally would benefit from a method to validate genotype data-sets and test the reliability of the designed primers. Levels of heterozygosity are reported for the prosobranch mollusc *Hydrobia ulvae *(Pennant) together with a method of genotype validation and primer assessment that utilises two primer sets for each locus. Microsatellite loci presented are the first described for the species *Hydrobia ulvae*; the five loci presented will be of value in further study of populations of *H. ulvae*.

**Results:**

We have developed a novel method of testing primer reliability in naturally heterozygote deficient populations. After the design of an initial primer set, genotyping in 48 *Hydrobia ulvae *specimens using a single primer set (Primer set_A) revealed heterozygote deficiency in six of the seven loci examined. Redesign of six of the primer pairs (Primer set_B), re-genotyping of the successful individuals from Primer set_A using Primer set_B, and comparison of genotypes between the two primer sets, enabled the identification of two loci (*Hulv-06 *&* Hulv-07*) that showed a high degree of discrepancy between primer sets A and B (0% & only 25% alleles matching, respectively), suggesting unreliability in these primers. The discrepancies included changes from heterozygotes to homozygotes or vice versa, and some individuals who also displayed new alleles of unexpected sizes. Of the other four loci examined (*Hulv-01*, *Hulv-03*, *Hulv-04*, &* Hulv-05*), all showed more than 95% agreement between primer sets. *Hulv-01*, *Hulv-03*, &* Hulv-*05 displayed similar levels of heterozygosity with both primer sets suggesting that these loci are indeed heterozygote deficient, while *Hulv-08 *showed no deficiency in either primer set.

**Conclusion:**

The simple method described to identify unreliable markers will prove a useful technique for many population studies, and also emphasises the dangers in using a single primer set and assuming marker reliability in populations shown to naturally deviate from HWE.

## Background

In recent years, the discovery and development of genetic markers such as microsatellites has led to a rapid growth in the number of molecular studies, with the isolation and development of microsatellite loci becoming relatively quick and straight-forward in many taxa [reviewed in [1]]. The method of Armour *et al*. 1994 has been successfully used for birds [2,3], mammals [4,5] and fish [6] but this success has not been ubiquitous across all taxa and the identification of microsatellite markers in many invertebrates has proved to be difficult, e.g. mosquitoes [7] and butterflies [8,9]. Even after successful microsatellite isolation, primers developed for certain species suffer from multiple banding patterns and random primer binding, leading to difficulties in obtaining reliable genotype data. The presence of repeat regions in the genome, known as SINEs (short interspersed nuclear elements), repeat microsatellite regions, and a general failure to amplify a specific product, have often been cited as potential causes for inaccuracies in data sets, for example in nematodes [10], lepidopterans [8,9,11] and marine molluscs [12-14].

In many marine invertebrates the situation is further complicated by a deficiency in the number of heterozygotes observed (relative to Hardy-Weinberg expectation) (see Additional file 1), with both allozyme and microsatellite studies documenting the phenomenon in many marine bivalve and gastropod populations [14-21]. While some of the species presented in Additional file 1 are hermaphroditic, e.g. *Physa acuta*, and thus may be expected to show heterozygote deficiency (due to high potential for selfing which would increase homozygosity in the population), there are many more examples (see Additional file 1) where species display separate sexes and would not necessarily be expected to display heterozygote deficiency.

With this in mind, one should consider the numerous factors which can cause heterozygote deficiencies. These include poor primer design and optimisation, null alleles [22], genotyping errors (stuttering or large allele dropout [23]), mutation, inbreeding effects, SINEs, non-random mating and population admixture. However, despite extensive examination in some cases [17,24] the causes behind these heterozygote deficiencies still remain unknown and, as a consequence, the ability to utilise traditional techniques for primer and population assessments may be significantly compromised.

In "model" populations, which are expected to conform to HWE, testing for heterozygote deficiency in loci is commonly used as a means to assess designed primers and genotype reliability. In such cases identification of an unreliable primer pair, or a locus affected by null alleles, is simply a matter of identifying heterozygote deficiency at that locus. This task is becoming increasingly simple due to the wide array of tools available for population and locus assessment (e.g. GENEPOP[25]; CERVUS[26]; MICROCHECKER, [27]). Once identified, the primer pair can then be removed from further study. Difficulties can arise, however, when attempting to identify unreliable primers and null alleles in populations that naturally display heterozygote deficiency (see Additional file 1). In these cases, the use of traditional analysis and software are no longer effective, as they often assume that populations conform to HWE and display expected heterozygosity. Thus, identifying unreliable primers by analysing departures from HWE is ineffective due to the fact that the population itself is naturally deficient. In these cases primer reliability is normally assumed without additional investigation beyond these standard tests (Additional file 1). As a result, unreliable primers may often remain undetected.

In this study microsatellite loci were isolated and examined for heterozygote deficiency in the prosobranch mollusc *Hydrobia ulvae*. A previous study by Haase [16] using allozyme markers suggests that *H. ulvae *populations are naturally heterozygote deficient; therefore further testing was conducted here to see if the same conclusions would be supported by microsatellite genotyping data. A novel technique was then used to assess microsatellite marker reliability by designing two microsatellite marker primer sets to amplify the same loci. By altering the binding sites, designing additional primers (B-primers) to amplify the same loci as the original primer set (A-primers), and comparing genotypes between A and B, a method is presented to assess the reliability of Primer set_A. Additionally, the method can also be used to identify inaccuracies due to PCR amplification failure and erroneous genotype scoring.

*Hydrobia ulvae *is a widespread and abundant member of the benthic fauna of estuarine habitats. It is dioecious with sexes being easily identified through dissection. On the west coast of Wales this species has peaks of spawning activity in spring and autumn and produces planktotrophic larvae that remain in the plankton for up to four weeks before settlement [28]. This period of development affords the potential for dispersal to new habitats and mixing with geographically separate populations. The species provides an interesting case for molecular analysis as the pelagic dispersal phase raises fascinating questions on gene flow, differentiation, recruitment, and inbreeding, but there remains the potential for self-recruitment of estuarine populations [29].

The objectives of this study were: (1) to isolate polymorphic microsatellite loci for *Hydrobia ulvae*, (2) to conduct standard tests for HWE, heterozygosity, linkage equilibrium and sex linkage and (3) to assess the use of alternative primer sets for the same loci as a tool to assess primer reliability.

## Results

### Microsatellite library preparation, Primer set_A design and testing

The first step was to isolate polymorphic microsatellite loci for *H. ulvae *and test to see if the population examined displayed heterozygote deficiency. Samples were collected from a presumed single population of *Hydrobia ulvae *from an area of approximately 1.0 m^2 ^in the Dyfi estuary, Wales (52° 31' 31.9" N; 4° 2' 40.2" W) using a 0.5 mm mesh sieve to remove individuals from the sediment. Specimens were stored alive in sea water of 28 ‰ at 14°C until required for DNA extraction.

Prior to DNA extraction, *Hydrobia ulvae *individuals were dissected and checked for parasitic infection. Those that showed signs of infection were removed from further study to avoid the possibility of isolating microsatellites from the parasites. Extraction of *Hydrobia *DNA and preparation of an enriched library yielded 118 unique microsatellite sequences that were submitted to the EMBL Nucleotide Sequence Database [30] [EMBL AM409397–AM409514]. For details of DNA extraction and enriched library development, see Methods. All the obtained sequences were confirmed to be unique using BLASTN version 2.2.4 [31]. Forty-seven loci possessed sufficient flanking sequence for primer design (in terms of repeat length, sequence length and base-pair composition of the flanking sequence). Primer pairs were designed for 17 loci using PRIMER3 [32], labelled with a fluorescent dye, and optimised with respect to temperature (gradient range 50–70°C) and MgCl_2 _concentration (1.5 mM, 2.0 mM and 2.5 mM). A short chain of nucleotides (GTTTCTT), known as a pigtail, was also added to the 5' end of each reverse primer to reduce stutter bands during PCR amplification [33].

The subsequent PCR and genotyping of 16 snails from the Dyfi estuary (including the six individuals from which the library was made) using 17 designed primers, resulted in nine primers failing to amplify a specific product, and one locus, *Hulv-10 *(AM409406; Forward-TCGTACCAGGAAAGGCCTCAG, Reverse-CCACGTCACTTTCGGTGCTC) being monomorphic in all 16 individuals tested. For each of the seven remaining polymorphic loci a further 48 *Hydrobia ulvae *individuals were genotyped. The primers designed for these seven loci are hereafter described as Primer set_A (Additional file 2). The expected and observed heterozygosity of each locus (as amplified with Primer set_A) was calculated and null allele frequency estimates obtained using CERVUS version 2.0 [26], while deviations from HWE and linkage equilibrium were examined using exact tests in GENEPOP (v3.4) [25]. Results showed that the seven polymorphic loci (when amplified using Primer set_A) were highly variable, with the number of alleles ranging from 16 to 27 (Additional file 2). Six of the loci were found to display a heterozygote deficiency when compared with the expectation under HWE. The exception was locus *Hulv-04_ A*, which was in Hardy-Weinberg equilibrium (Additional file 2). Two of the loci (*Hulv-06 *and *Hulv-07*) showed very low levels of heterozygosity, suggesting possible unreliability in the primers (Additional file 2). However, to justify the removal of these loci and designed primers, further examination was required, as low heterozygosity is not necessarily a precursor for unreliable markers. Another notable result was the amplification success shown by *Hulv-01_A*, *Hulv-06_A *and *Hulv-07_A *primers. At these loci, amplification success was slightly lower than expected (83.3%, 83.3% and 70.8% of individuals amplified, respectively). Given that high levels of amplification failure can be used as an indicator of unreliable primers, the opportunity was taken to explore the connection between amplification failure and primer reliability by examining these loci using the primer redesign technique described.

As well as heterozygote analysis, tests were conducted using the software MICROCHECKER[27] to examine genotyping error, presence of null alleles, allele frequency estimates, allele stutter and allele dropout (in which smaller alleles are preferentially amplified over larger alleles [23]). Interestingly, all results were negative except for null alleles, which the software highlighted could be a potential factor in causing the heterozygote deficiency at all but one of the loci, *Hulv-04*. For this reason, and to ensure that alleles were not failing to amplify due to the PCR conditions, each locus was PCR-amplified twice using each primer set (A & B). On both occasions the same genotypes were observed. Alleles were resolved on an agarose gel and allele sizes were checked to see if they were consistent with the size expected based on the sequence of the cloned allele. Examination of the gel showed no indication of large alleles (> 500 bp), suggesting that neither SINE insertions, nor duplicate microsatellite regions, were present in the products. Pedigrees were not available for checking the inheritance of null alleles due to difficulties in cultivating the species in the laboratory.

Loci were also examined for sex linkage by visually comparing the genotypes of individuals of known sex (sexes assigned after dissection), and the results suggested no association between apparent homozygosity and sex in the genotyped individuals. Similarly, all loci were checked for linkage disequilibrium using GENEPOP v3.4 without Bonferroni correction and no linkage disequilibrium was observed.

### Comparing genotype data from two sets of primers for each locus

In order to test genotype outputs and Primer set_A reliability, microsatellite primers were redesigned (Primer set_B) (Additional file 3) for six loci (*Hulv-01*, *Hulv-03*, *Hulv-04*, *Hulv-05*, *Hulv-06 *&* Hulv-07*) to amplify the same loci as Primer set_A. "B" primers were not designed for *Hulv-02 *due to difficulty in identifying new primer sites (due to the length and base-pair composition of the flanking sequence), so this locus could not be verified. In all other cases, primers were designed at sites as distinct as possible from the original primer sites. In some cases, short flanking regions hindered primer design and thus only one of the primers in a pair could be significantly shifted. However, where possible the 3' end of the primer was redesigned so that primer reliability could still be assessed. Single mismatches at or near the 3' end of the primer are known to affect the efficiency of polymerase extension and oligonucleotide stability [34] and will have a greater effect upon amplified products than mismatches elsewhere [35,36], thus where possible an alteration in the 3' end of the primer sequence was attempted. The relevance of this restriction in the current study and to the technique as a whole is discussed later.

Redesigned Primer set _B was optimised, amplified and visualised in exactly the same way as Primer set_A, using only the DNA templates from individuals which yielded successful amplification in Primer set_A. Samples which failed to amplify a product in Primer set_A were not examined using Primer set_B, as regardless of the output from Primer set_B in these cases, no comparison could ever be made. This was also done so that direct comparison of successful amplifications could be made between the primer sets. Alleles were scored independently on separate ABI3730 gel loading runs for each primer set (A and B) to prevent any bias in the data.

To assess primer data, the presence and size of alleles from Primer set_B were compared with those originally amplified by Primer set_A. Allele sizes were expected to show a consistent and predictable difference in size between the primer sets within each locus, due to the difference in product size caused by the redesign and movement of primers. To be classified as a successful match between primers, both alleles in a heterozygote needed to be the same for both primer sets. Likewise, in homozygotes the single alleles needed to display the same product after the predictable size difference was taken into account.

Results indicate that when both primer sets (A and B) were used in conjunction to amplify the six target loci, four of these (*Hulv-01*, *Hulv-03*, *Hulv-04*, &* Hulv-05*) showed more than 95% agreement (allele-allele) between the alleles amplified by the two primer sets A and B (Additional file 4), suggesting that these primers from Primer set_A had amplified the target loci correctly, and thus are reliable. However, when comparison was made between Primer set_A and Primer set_B for the remaining two loci (*Hulv-06 *&* Hulv-07*), a high degree of discrepancy was observed (0% & only 25% alleles matching, respectively) (Additional file 4). These discrepancies included a change from heterozygotes to homozygotes or vice versa, while some samples also displayed new unexpected allele sizes. Samples which failed to amplify the same target loci in each primer set were examined to ensure that poor DNA was not the underlying cause. No individuals consistently failed to amplify and the individuals failing to amplify differed between loci and thus poor DNA samples were not to blame for amplification failures.

When these findings are coupled with the previously identified decreased amplification success at these loci, it is clear that *Hulv-06_A *and *Hulv-07_A *primers are not suitable for further use in population studies due to their unreliability. Interestingly, despite the fact that *Hulv-01_A *was previously shown to display similar levels of amplification failure as *Hulv-06_A *and *Hulv-07_A*, analysis using redesigned markers has highlighted that *Hulv-01_A *primers were in fact producing reliable genotype data. Therefore the redesign technique presented here demonstrates that decreased amplification success does not necessary mean a marker is unreliable.

## Discussion

This study has shown that in order to identify and confidently remove unreliable markers in heterozygote deficient populations, additional techniques are required beyond those currently applied to microsatellite data. The currently used tests such as examination of HWE, heterozygosity, amplification failure, allele frequency distributions and linkage equilibrium are not sufficient to identify reliability in primer sets. This may particularly be the case for invertebrate species, many of which commonly display departures from HWE and heterozygote deficiencies [14,19]. The results show that the majority of the loci examined (6/7) displayed heterozygote deficiency (Additional file 2) and *H. ulvae *therefore exhibits similar characteristics to many of the marine and freshwater invertebrates (as detailed in Additional file 1). The technique presented here has shown that after primer redesign, *Hulv-06 *and *Hulv-07 *cannot be reliably genotyped due to the discrepancies between each primer set. Therefore it is concluded that these loci, along with designed primers, should not be used in future studies. Similarly, the technique has also provided increased confidence in the remaining loci (*Hulv-01*, *Hulv-03*, *Hulv-04 *and *Hulv-05*), which all showed similar genotype outputs regardless of the primer set implemented. For this reason, the technique described here presents a useful method for helping to assess the reliability of designed primers in heterozygote-deficient populations.

Previous published studies have suggested many causes for heterozygote deficiency in invertebrates, including selection, inbreeding, mutation and null alleles, SINEs, poor primer design and amplification error [14,19]. However, attempts to ascertain the exact source of the heterozygote deficiency have often had limited success [21,24], and while it is not the purpose of this study to identify the source of the heterozygote deficiency, the technique and results obtained can be used to shed light into the cause of the heterozygosity in the species.

The first factor to consider is selection. While some microsatellite loci have been shown to be affected by selection [37,38], the majority are still considered to be selectively neutral [39]. This study took care not to incorporate tri-nucleotide repeats, which have been shown to occur in coding regions of the genome and thus are more likely to be subject to selection [40]. Therefore, while it cannot be categorically ruled out, selection is not likely to be the cause of the observed deficiencies. Similarly, given what is known about the life stages of the target organism, inbreeding seems unlikely. In species with limited dispersal or direct development, it is simple to imagine inbreeding as a dominant force [41,42], which can be detrimental to species fitness [43,44]. However, given that *Hydrobia ulvae *has a dispersal phase of up to four weeks [28], a good potential for mixing of progeny by tides and currents, and the likelihood of widespread settlement of larvae within the estuary, it seems highly improbable that inbreeding is occurring. Another alternative potential cause is the Wahlund effect, which is a heterozygote deficiency due to the accidental pooling of discrete sub-populations. Indeed, by disregarding sub-structuring within populations one would expect to see a common deficiency across the majority of the loci as observed in this study [14,42]. However, it is unlikely that samples taken from a homogeneous area of less than 1 m^2^, as done here, could contain many different sub-populations [16]. Nonetheless, in order to be certain, additional genotypic analysis would be required on individuals from identified cohorts and known local spatial localities [41].

Perhaps the most interesting factor to consider with regard to the technique presented here is the presence of null alleles and their potential effect upon levels of heterozygosity. Null alleles represent base-pair mutations in the primer regions which cause primer binding to weaken and/or fail, resulting in a failure to amplify certain alleles [45]. As a result, the presence of null alleles in data sets has been commonly suggested as a contributor to heterozygote deficiency [46,47] (Additional file 1). While microsatellite regions are often highly polymorphic due to a high rate of mutation through replication slippage and proof-reading events [48], the flanking regions surrounding microsatellite repeat regions are generally considered to be more conserved. However, given the very high levels of polymorphism shown in microsatellite loci examined for *H. ulvae *(Additional file 2), it is possible that the sequence flanking the repeat regions may also exhibit increased levels of mutation which would certainly reduce the effectiveness of primer binding and result in an abundance of null alleles. Microsatellite loci in humans have been estimated to have mutation rates of about 10^-4 ^[49]. However, microsatellite mutation is known to vary between different taxa [50,51], and while little is known specifically about the mutation rate in marine molluscs, several studies, including this current study, have shown high polymorphism in microsatellite loci in marine invertebrates [14,20,52], suggesting that mutation rates may be high. For this reason further investigation in marine invertebrates is required (i.e. genetic sequencing) to determine whether mutations in the flanking regions introduce errors into genotype data and consequently influence levels of heterozygosity.

Despite this, there are several reasons to suggest that mutation and null alleles are not the explanation for the overall heterozygote deficiency observed in *Hydrobia ulvae*. First, given their nature one would typically expect null alleles to occur at a minority of loci and not across the majority of loci as seen in this study [14,19,39]. Secondly, results from the double-primer technique show similar heterozygote deficiencies and null allele frequencies in both primer sets A and B in all loci with the exception of *Hulv-06 *and *Hulv-07 *(Additional file 4). If nulls were the explanation for the heterozygote deficiency then we would not expect both primer sets to be equally affected.

Mutation and null alleles do however, present one possible explanation for the poor match between primer sets for *Hulv-06 *and *Hulv-07*, particularly as the predicted null allele frequencies at these loci were shown by the software MICROCHECKER to be much higher than for all the other loci examined (Additional file 4). Indeed, when coupled with the low amplification rate of individuals shown by *Hulv-06_A *and *Hulv-07_A *primers (83.3 and 70.8%, respectively), there is evidence to suggest mutation in the flanking regions and null alleles. Alternatively, the mismatch between primer sets A and B for *Hulv-06 *and *Hulv-07 *could be due to similarity in the flanking regions of different loci. In the study by Meglecz *et al. *[8] on lepidopterans (butterflies and moths), high flanking sequence similarity was observed at a number of loci, which led to difficulties in designing effective primer sets. Likewise, in a study on the marine gastropod *Littorina saxatilis*, anomalous large alleles were identified that may signify the presence of flanking similarity in marine invertebrates [20]. While no evidence has been found in the present study for large alleles above 500 bp as described by Sokolov *et al. *[20], it is entirely possible that flanking region similarity could introduce errors into genotyped data and thus disrupt the levels of heterozygosity observed. However, while further investigation into the matter is required, the fact that very little sequence similarity was identified in this study suggests that it is not a dominant factor.

While the precise nature of the overall heterozygote deficiency still remains unclear, it is concluded that mutation and null alleles are the likely explanation for the unreliability observed at two of the loci examined (*Hulv-06 *and *Hulv-07*). Drawing definitive conclusions on the presence of null alleles, the cause of heterozygote deficiency and primer reliability, has been limited in this study due to constraints on primer redesign imposed by the restricted length of flanking regions. However, when redesigning primers, attempts were made to make B-primers as distinct from A-primers as possible, particularly at the 3' end of the primer, as this region is known to affect product amplification and polymerase extension [34,36,35]. Given that the occurrence of restricted flanking regions in microsatellite studies has not been commonly reported, and that information regarding the length of flanking regions in different species is scarce, it is difficult to estimate the likelihood of primer redesign complications in other species; nevertheless, it is believed that the technique presented here provides an important contribution to the field, as it offers a practical alternative to merely assuming primer reliability in heterozygote-deficient populations. Most importantly, this study has highlighted the need for increased caution in assessing primer reliability using only standard assessments, especially in populations commonly exhibiting heterozygote deficiency and deviations from HWE. Moreover, this study has illustrated that additional testing of designed primers in heterozygote deficient populations can identify potentially unreliable markers, amplification errors and genotyping failures, which are commonly associated with microsatellite studies [12,39]. For these reasons, the methodology presented will be of potential interest/application to future studies on invertebrate and non-invertebrate species alike. Indeed, the technique will be of value in all microsatellite studies that seek increased confidence in genotype assignments. While doubling expenditure on primers may be seen as a disadvantage, the benefits make the technique a sound investment when one considers the high cost of running unreliable primers in large-scale population analysis. The technique will also be of particular use in studies where small primer sets are used due to difficulties in microsatellite isolation. In these cases, it is imperative that all primers consistently amplify a reliable product, given the low number being implemented. Similarly, the genotype data validation provided by this technique will also enable increased confidence in species where multiple banding patterns and anomalous large alleles are often noted [20].

In addition to the technique proposed, five novel *Hydrobia ulvae *polymorphic loci (*Hulv-01*, *Hulv-02*, *Hulv-03*, *Hulv-04 *&* Hulv-05*) (Additional file 2) are presented for further use in population studies. Given their level of polymorphism, these microsatellites, and the primers described, will provide valuable tools in the study of genetic mixing and population differentiation in *Hydrobia ulvae*.

## Conclusion

While it is clear that *Hydrobia ulvae *is characteristic of its taxon with regard to heterozygosity levels, the methods described here provide a useful tool for assessing genotype data and primer reliability in studies where increased confidence in microsatellites is desired. While traditional examination of HWE and heterozygote deficiency has been shown to be insufficient to identify unreliable primers in naturally deficient populations, the use of dual primers provides a simple alternative to merely assuming reliability. In studies that have previously only used traditional methods, or assumed primer reliability, the technique may serve to identify unreliable primer pairs early in the study before the cost of genotyping multiple individuals is incurred.

Therefore, while it is clear that standard examinations and software can serve to highlight many primer reliability issues in model populations, heterozygote deficient and other non-model populations require the additional validation provided by designing a second primer set. In order for microsatellite primers to be used effectively in population studies and to justify the lengthy developmental process, they need to be reliable and of sufficient power, a situation which is more likely when validation steps are taken.

## Methods

### DNA isolation

Genomic DNA was extracted for microsatellite library development and genotyping using a modified CTAB procedure with proteinase K digestion and a chloroform-isoamylalcohol protocol [53].

### Microsatellite library preparation

An enriched *Hydrobia ulvae *microsatellite library was prepared using a method based on Armour *et al. *[54], with modifications described by Gibbs *et al. *[55]. Six *Hydrobia ulvae *individuals of unknown sex were pooled to obtain a sufficient amount of genomic DNA from which to prepare the library. The pooled genomic DNA (2 μg) was digested using *Mbo*I (Qbiogene, Cambridge, UK), and ligated to double-stranded Sau-L linkers [56]. Size-selected (200–800 bp), digested genomic DNA fragments were then denatured and hybridised against double-stranded denatured dinucleotide and tetranucleotide sequences that had been bound to nylon Hybond membrane (Amersham Pharmaceuticals Ltd, Buckinghamshire, UK). The Armour [54]*et al. * pre-enrichment PCR was not performed [as in [55]]. The dinucleotide sequences (AC.GT)_n _and (AG.CT)_n _were obtained as DNA Alternating Copolymers (Amersham Pharmaceuticals Ltd, Buckinghamshire, UK) and the tetranucleotide sequences (TTTC.GAAA)_n_, (GTAA.TTAC)_n_, (GATA.TATC)_n_, (CTAA.TTAG)_n _and (TAAA.TTTA)_n _were prepared using two rounds of PCR amplification as in Armour *et al. *[54]. Once recovered, the microsatellite-enriched *Hydrobia ulvae *fragments were separated from the Sau-L linkers by digestion with *Mbo*I. Fragments were then ligated into *Bam*HI/BAP-dephosphorylated pUC19 vector (Qbiogene) and transformed into XL1-Blue competent cells (Stratagene). Transformant colonies were grown overnight at 37°C on agar plates containing Luria Broth, Ampicillin, X-gal and IPTG, bound to a Hybond nylon filter and screened with [α^32^P]-dCTP and/or [α^32^P]-dATP radiolabelled dinucleotide and tetranucleotide sequences as described above. In total, 902 transformants were screened and 265 produced a positive autoradiograph signal (4 of 18 from the dinucleotide library and 261 of 884 from the tetranucleotide enriched library). Sequence data for 153 clones was obtained using an ABI 3730 capillary sequencer with BigDye chemistry (Applied Biosystems). All sequences were checked for duplication using stand-alone BLAST software (protocol available at [57] and MEGA v.3.1 sequence alignment software [58]. Of the 153 sequences examined, 118 were found to be unique.

### PCR and genotyping

When genotyping individual *Hydrobia ulvae*, each PCR reaction contained approximately 5–10 ng of genomic DNA, 0.5 μM of each primer, 1.5–2 mM MgCl_2 _(Additional file 2), 0.2 mM of each dNTP and 0.05 units of *Taq *DNA polymerase (Bioline, London, UK) in the manufacturer's buffer [final concentration: 16 mM (NH_4_)_2_SO_4_, 67 mM Tris-HCl (pH 8.8 at 25°C), 0.01% Tween-20]. PCR amplification was performed using a thermal cycler (MJ Research model PTC DNA engine) with the following program: one cycle of 3 min at 94°C, followed by: 35 cycles of 94°C for 30 s, annealing temperature (Additional file 2) for 30 s, 72°C for 45 s and a final extension cycle of 10 min at 72°C. PCR products were visualised on 2% agarose gel, pre-stained with ethidium bromide. Amplified product sizes were compared to the size expected based on the cloned allele sequence and checked for the presence of large alleles (> 500 bp), a potential by-product of SINE insertions [8,9].

A fraction of the fluorescently-labelled PCR product was diluted to one part per thousand and loaded on an ABI 3730 DNA Analyzer, along with ROX500 size marker (Applied Biosystems). Allele sizes were assigned using GENEMAPPER 3.7 software (Applied Biosystems).

## Authors' contributions

RJB collected and processed specimens for examination, carried out the molecular genetics studies and drafted the manuscript. DAD supervised the molecular work, assisted with interpretation of data and assisted in drafting of the manuscript. GJH prepared the microsatellite-enriched genomic library. JJB contributed to the initial design of the project. JDF conceived the study, and participated in its design and coordination and helped to draft the manuscript. All authors read and approved the final manuscript.

## Supplementary Material

Additional file 1 Review of heterozygosity in microsatellite studies of marine and freshwater gastropods. Click here for file

Additional file 2 Characterisation of seven polymorphic microsatellite loci (Primer set_A) for *Hydrobia ulvae*. Click here for file

Additional file 3 Characterisation of redesigned Primer set_B for *Hydrobia ulvae*. Click here for file

Additional file 4 Comparison of microsatellite genotyping data using two primer sets (A and B) for *Hydrobia ulvae*. Click here for file
