# Supplementary material for: A method for genotype validation and primer assessment in heterozygote-deficient species, as demonstrated in the prosobranch mollusc Hydrobia ulvae
Source: BMC Genet. 2008 Aug 19;9:55. doi: 10.1186/1471-2156-9-55 (PMC2536670; doi:10.1186/1471-2156-9-55)
Supplement: Additional file 1 — Review of heterozygosity in microsatellite studies of marine and freshwater gastropods. [file 1471-2156-9-55-S1.pdf]

Additional file 1. Review of heterozygote deficiency in microsatellite studies of marine and freshwater gastropods

Species	Common name	Reproductive method	Evidence of selfing?	No. of loci identified	Presence of heterozygote deficient loci (number, if stated)	Suggested cause of heterozygote deficit	Reference	Notes
Biomphalaria pfeifferi	Freshwater snail	Hermaphroditic	Yes	9	Yes	Self-fertilisation	Charbonnel *et al.*(2000)	-
Buccinum undatum	Common whelk	Separate sexes	No	5	Yes (2/5)	Null alleles	Weetman *et al.*(2005)	Poor primer success (only five loci successful from 38 designed)
Bulinus forskalii	African freshwater snail	Hermaphroditic	Yes	11	Yes (11/11)	High degree of selfing	Gow *et al.*(2001)	-
Bulinus globosus	Freshwater snail	Hermaphroditic	Yes	6	Yes (4/6)	Null alleles (described as unlikely as no there were no consistently failing amplifications at any single loci), population bottleneck effect, and self-fertilisation	Emery *et al.*(2003)	-
Cerithium lividulum	Needle whelk	Separate sexes	No	6	Yes (1/6)	Null alleles (described as unlikely as no there were no consistently failing amplifications at any single loci), population bottleneck effect, and self-fertilisation	Emery *et al.*(2003)	-
Cerithium vulgatum	Needle whelk	Separate sexes	No	6	Yes (4/6)	Population-level processes and small sample sizes	Samadi *et al.*(2001)	-
Crepidula fornicata	Slipper limpet	Protandrous, hermaphrodite	Yes	7	Yes	Null alleles (described as unlikely as observed in multiple populations with variable intensities across the loci), large number of rare alleles and the Wahlund effect	Dupont & Viard (2003)	-
Cyclope neritea	Gastropod mollusc	Separate sexes	No	8	Yes	Null alleles due to phylogeographical break in the species' native range	Simon-Bouhet *et al.*(2005)	-
Haliotis kamtschatkana	Northern abalone	Separate sexes	No	12	Yes (6/12)	Null alleles (described as unlikely due to presence over majority of loci), non-random mating, inbreeding, temporal variation in the genetic composition of recruits, or population admixture	Miller *et al.*(2001)	-
Haliotis midae	Perlemoen abalone	Separate sexes	No	11	Yes (4/11)	Null alleles	Bester *et al.*(2004)	Suffered from allele dropout effect that could not be resolved through optimisation
Haliotis rubra	Black-lip abalone	Separate sexes	No	9	Yes	No explanation offered	Evans *et al.*(2000)	
Littorina saxatilis	Rough periwinkle	Separate sexes	No	7	Yes	Null alleles (described as unlikely as did not demonstrate presence of null homozygotes i.e. complete failures), pooling of individuals from several sub-populations (Wahlund effect)	Sokolov *et al.*(2002)	Described large anomalous alleles (500-700 bp)
Littorina striata	Periwinkle	Separate sexes	No	5	Yes (3/5)	Null alleles (described as unlikely due to presence over majority of loci), limited sample size and population sub structuring	Winnepenninckx & Backeljau (1998)	-
Littorina subrotundata	Periwinkle	Separate sexes	No	12	Yes	No explanation offered	Tie *et al.*(2000)	-
Lymnaea stagnalis	Great pond snail	Hermaphroditic	Yes	9	Yes	Null alleles	Knott *et al.*(2003)	-
Lymnaea truncatula	Dwarf pond snail	Hermaphroditic	Yes	9	Yes (9/9)	Bottleneck effect and inbreeding	Trouve *et al.*(2000)	-
Melongena corona	Crown conch	Separate sexes	No	8	Yes	Null alleles (described as unlikely as did not demonstrate presence of null homozygotes i.e. complete failures) and template DNA	Hayes & Karl (2004)	-
Nucella lapillus	Dog whelk	Separate sexes	No	14	Yes	Population subdivision or inbreeding	Kawai *et al.*(2001)	-
Physa acuta	Freshwater snail	Hermaphroditic	Yes	7	Yes	Null alleles (described as unlikely due to nature of target species and consistent heterozygote deficit over multiple localities and loci)	Monsutti & Perrin (1999)	-
