# Supplementary material for: A method for genotype validation and primer assessment in heterozygote-deficient species, as demonstrated in the prosobranch mollusc Hydrobia ulvae
Source: BMC Genet. 2008 Aug 19;9:55. doi: 10.1186/1471-2156-9-55 (PMC2536670; doi:10.1186/1471-2156-9-55)
Supplement: Additional file 2 — Characterisation of seven polymorphic microsatellite loci (Primer set_A) for Hydrobia ulvae. [file 1471-2156-9-55-S2.pdf]

Additional file 2. Characterisation of seven polymorphic microsatellite loci (Primer set_A) for *Hydrobia ulvae*

Locus	Clone name	Primer set	EMBL accession number	Repeat motif	Exp. allele size (bp) †	Primer sequence 5'–3' with 5' label or pigtail* (underlined)‡	*Pv3* T_m (°C)Ψ	T_a (°C)	$MgCl_2$ (mM)	*N*	AS % (N/48* 100)	*A*	Allele range (bp)	H_O	H_E	HWE *p*-value
Hulv-01	66C08	A	AM409397	$GA(CA)_3(GACA)_4(GA)_2(CA)_2(GA)_{22}CA(GA)_7CA(GA)_4CA(GA)_4CA(GA)_7$	210	F: HEX-CACACAAATCACATGTCAGTTCAATAC<br>R: (GTTTCTT)-GAGAAGGATTGTCTACAGAAGG	61.8<br>61.6	55.0	2.0	40	83	25	194 – 318	0.65	0.84	**
Hulv-02	67G01	A	AM409398	$GTTAGTTG\ (GTTA)_3(GTCA)_{14}(GTTA)_8\ GTTGGTTA\ (GATA)_2\ GTTGGTTA$	217	F: HEX-GCGATAAACCACTTGTAGGG<br>R: (GTTTCTT)-TCTATCTAACCAACTATCTATCT AACCAAC	57.3<br>57.4	58.0	2.0	47	98	18	170 – 250	0.68	0.87	**
Hulv-03	67H10	A	AM409399	$(A)_{23}$ & $CTTACTA(CTTA)_7$	203	F: HEX-CGTCCCACTCTCGATGAAGC<br>R: (GTTTCTT)-TCAATAGGGAAGGGGTGAGTCC	63.3<br>63.6	64.0	2.0	46	96	16	186 – 208	0.59	0.89	**
Hulv-04	68D02	A	AM409400	$(CA)_{30}AACA$	256	F: 6-FAM-TCAATAAAATGAAGTTGAGTTGAG<br>R: (GTTTCTT)-GATCTGCAAGAGAGATGTAAGAA	55.5<br>55.5	51.0	2.0	47	98	27	218 – 288	0.92	0.91	NS
Hulv-05	69C07	A	AM409401	$(GTTA)_{21}$	249	F: 6-FAM-GATCGTAGGCTACTTACTACCACTG<br>R: (GTTTCTT)-TTCGTTGTTTCCCGTCTG	65.3<br>65.1	64.0	1.5	44	92	21	188 – 320	0.57	0.93	**
Hulv-06	60F03	A	AM409402	$(CTAA)_4(CTCA)_{12}$	185	F: HEX-GATCCACAAAGCAACAGCTATAACTAA<br>R: (GTTTCTT)-AACACGGACATGAACACTGGA	61.1<br>61.4	58.0	2.0	40	83	17	135 – 215	0.20	0.93	**
Hulv-07	69A07	A	AM409403	$(GTTA)_9$	124	F: HEX-AGCTTTGACTATTCTTTGTGAGC<br>R: (GTTTCTT)-GATCAATAATCGGGCTTAAAAC	57.2<br>56.9	51.0	2.0	34	71	19	106 – 202	0.38	0.94	**

† Allele size in sequenced library source individual; ‡Pigtail: GTTTCTT added to 5' end of reverse primer to reduce stutter bands [33].
Note that in all cases the 7 base pair pigtails have been incorporated in the allele sizes stated.
Ψ, Primers designed and melting temperature (T_m) calculated using PRIMER3 (*Pv3*) [32].
T_a, annealing temperature; *N,* number of individuals successfully genotyped from the Dyfi estuary, Wales; AS, percentage amplification success; *A,* number of alleles observed; H_O, observed heterozygosity; H_E, expected heterozygosity.
**Locus deviates significantly from Hardy-Weinberg equilibrium (p<0.01), estimated using GENEPOP v3.4 [25].
NS, no significant deviation from Hardy-Weinberg equilibrium.
