# Supplementary material for: A method for genotype validation and primer assessment in heterozygote-deficient species, as demonstrated in the prosobranch mollusc Hydrobia ulvae
Source: BMC Genet. 2008 Aug 19;9:55. doi: 10.1186/1471-2156-9-55 (PMC2536670; doi:10.1186/1471-2156-9-55)
Supplement: Additional file 3 — Characterisation of redesigned Primer set_B for Hydrobia ulvae. [file 1471-2156-9-55-S3.pdf]

Additional file 3. Characterisation of redesigned Primer set_B for *Hydrobia ulvae*

Locus	Corresponding A-primer clone name	Primer set	Repeat motif	Exp. allele size (bp) †	Primer sequence 5'–3' with 5' label or pigtail* (underlined)‡	*Pv3* T_m (°C)Ψ	T_a (°C)	$MgCl_2$ (mM)	*N*	*A*	Allele range (bp)	H_O	H_E	HWE *p*-value
Hulv-01	66C08	B	$GA(CA)_3(GACA)_4(GA)_2(CA)_2(GA)_{22}CA(GA)_7CA(GA)_4CA(GA)_4CA(GA)_7$	230	F: HEX-GATCTGGGGCACACACAA<br>R: (GTTTCTT)-CACAAATCGGGGAGAAGG	59.0<br>59.0	51.0	2.0	40	24	214 – 338	0.63	0.83	**
Hulv-02	67G01	B	Primers not redesigned due to difficulty in identifying new primer sites											
Hulv-03	67H10	B	$(A)_{23}$ & $CTTACTA(CTTA)_7$	172	F: HEX-GACTCGGGCACTCCACAGTAAA<br>R: (GTTTCTT)-TCAATAGGGAAGGGGTGAGTCC	63.7<br>63.6	55.0	2.0	46	16	155 – 177	0.59	0.89	**
Hulv-04	68D02	B	$(CA)_{30}AACA$	172	F: 6-FAM-ACGGGTGTAACAACGACAAT<br>R: (GTTTCTT)-GATCTGCAAGAGAGATGTAAGAACT	57.9<br>57.5	51.0	2.0	47	27	134 – 204	0.92	0.91	NS
Hulv-05	69C07	B	$(GTTA)_{21}$	235	F: 6-FAM-GATCGTAGGCTACTTACTACCACTG<br>R: (GTTTCTT)-TCTGACCTGGTGTGTCATGT	57.8<br>57.4	51.0	2.0	44	21	174 – 306	0.59	0.93	**
Hulv-06	60F03	B	$(CTAA)_4(CTCA)_{12}$	217	F: HEX-CACAAAGCAACAGCTATAACTAA<br>R: (GTTTCTT)-TGATGGTGTCGTACTGAATC	54.5<br>54.3	51.0	2.0	40	21	169 – 249	0.33	0.94	**
Hulv-07	69A07	B	$(GTTA)_9$	154	F: 6-FAM-CCGCACTACCAATGTAGC<br>R: (GTTTCTT)-ATCAATAATCGGGCTTAAAAC	55.2<br>55.1	50.0	2.0	34	20	123 – 236	0.12	0.95	**

†Allele size in sequenced library source individual; ‡Pigtail: GTTTCTT added to 5' end of reverse primer to reduce stutter bands [33].
Note that in all cases the 7 base pair pigtails have been incorporated in the allele sizes stated.
Ψ, Primers designed and melting temperature (T_m) calculated using PRIMER3 (*Pv3*) [32].
T_a, annealing temperature; *N,* number of individuals successfully genotyped from the Dyfi estuary, Wales; *A,* number of alleles observed;
H_o, observed heterozygosity; H_E, expected heterozygosity.
**Locus deviates significantly from Hardy-Weinberg equilibrium ($p<0.01$), estimated using GENEPOP v3.4 [25].
NS, no significant deviation from Hardy-Weinberg equilibrium.
