# Supplementary material for: A method for genotype validation and primer assessment in heterozygote-deficient species, as demonstrated in the prosobranch mollusc Hydrobia ulvae
Source: BMC Genet. 2008 Aug 19;9:55. doi: 10.1186/1471-2156-9-55 (PMC2536670; doi:10.1186/1471-2156-9-55)
Supplement: Additional file 4 — Comparison of microsatellite genotyping data using two primer sets (A and B) for Hydrobia ulvae. [file 1471-2156-9-55-S4.pdf]

Additional file 4. Comparison of microsatellite genotyping data using two primer sets (A and B) for *Hydrobia ulvae*

Locus	Primer set	H_E - H_O	Deviation from HWE	P-value of deviation from HWE	MICROCHECKER explanation of results	Predicted null allele frequency	Proportion of matching genotypes (%)	Potentially unreliable locus?
Hulv-01	A	0.19	Yes	<0.001**	Analysis indicates homozygote excess at this locus. No evidence for scoring error due to stuttering. No evidence for large allele dropout. Null alleles may be present at this locus, as is suggested by the general excess of homozygotes for most allele size classes.	0.12	38/40 (95%)	Yes
	B	0.20	Yes	<0.001**	(see *Hulv-01*_set A)	0.14		
Hulv-03	A	0.30	Yes	<0.001**	(see *Hulv-01*_set A)	0.20	46/46 (100%)	Yes
	B	0.30	Yes	<0.001**	(see *Hulv-01*_set A)	0.20		
Hulv-04	A	-0.01	No	0.738	No evidence for scoring error due to stuttering. No evidence for large allele dropout. No evidence for null alleles.	-0.01	47/47 (100%)	Yes
	B	-0.01	No	0.852	As for *Hulv-04*_set A	-0.01		
Hulv-05	A	0.36	Yes	<0.001**	(see *Hulv-01*_set A)	0.24	43/44 (98%)	Yes
	B	0.34	Yes	<0.001**	(see *Hulv-01*_set A)	0.22		
Hulv-06	A	0.73	Yes	<0.001**	(see *Hulv-01*_set A)	0.64	0/40 (0%)	No
	B	0.61	Yes	<0.001**	(see *Hulv-01*_set A)	0.48		
Hulv-07	A	0.56	Yes	<0.001**	(see *Hulv-01*_set A)	0.42	8/34 (24%)	No
	B	0.83	Yes	<0.001**	(see *Hulv-01*_set A)	0.78		

H_O, observed heterozygosity; H_E, expected heterozygosity; HWE, Hardy-Weinberg equilibrium.
**Locus deviates significantly from Hardy-Weinberg equilibrium ($p<0.01$), estimated using GENEPOP v3.4 [25].
